# Chemical evidence of inter-hemispheric air mass intrusion into the Northern Hemisphere mid-latitudes

**DOI:** 10.1038/s41598-018-22266-0

**Published:** 2018-03-16

**Authors:** S. Li, S. Park, J.-Y. Lee, K.-J. Ha, M.-K. Park, C. O. Jo, H. Oh, J. Mühle, K.-R. Kim, S. A. Montzka, S. O’Doherty, P. B. Krummel, E. Atlas, B. R. Miller, F. Moore, R. F. Weiss, S. C. Wofsy

**Affiliations:** 1 Kyungpook Institute of Oceanography, College of Natural Sciences, Kyungpook National University, Daegu, South Korea; 2 Department of Oceanography, School of Earth System Sciences, Kyungpook National University, Daegu, South Korea; 3 Center for Climate Physics, Institute for Basic Science, Busan, South Korea; 4 Research Center for Climate Sciences, Pusan National University, Busan, South Korea; 5 Department of Atmospheric Sciences, Pusan National University, Busan, South Korea; 6 Scripps Institution of Oceanography, University of California, San Diego, La Jolla, CA USA; 7 GIST College, Gwangju Institute of Science and Technology, Gwangju, South Korea; 8 Earth System Research Laboratory, NOAA, Boulder, CO USA; 9 School of Chemistry, University of Bristol, Bristol, UK; 10 Climate Science Centre, CSIRO Oceans and Atmosphere, Aspendale, Victoria, Australia; 11 Rosenstiel School of Marine and Atmospheric Science, University of Miami, Miami, USA; 12 Cooperative Institute for Research in Environmental Sciences, University of Colorado, Boulder, Colorado USA; 13 School of Engineering and Applied Sciences, Harvard University, Cambridge, MA USA

## Abstract

The East Asian Summer Monsoon driven by temperature and moisture gradients between the Asian continent and the Pacific Ocean, leads to approximately 50% of the annual rainfall in the region across 20–40°N. Due to its increasing scientific and social importance, there have been several previous studies on identification of moisture sources for summer monsoon rainfall over East Asia mainly using Lagrangian or Eulerian atmospheric water vapor models. The major source regions for EASM previously proposed include the North Indian Ocean, South China Sea and North western Pacific. Based on high-precision and high-frequency 6-year measurement records of hydrofluorocarbons (HFCs), here we report a direct evidence of rapid intrusion of warm and moist tropical air mass from the Southern Hemisphere (SH) reaching within a couple of days up to 33°N into East Asia. We further suggest that the combination of direct chemical tracer record and a back-trajectory model with physical meteorological variables helps pave the way to identify moisture sources for monsoon rainfall. A case study for Gosan station (33.25°N, 126.19°E) indicates that the meridional transport of precipitable water from the SH accompanying the southerly/southwesterly flow contributes most significantly to its summer rainfall.

## Introduction

The East Asian summer monsoon (EASM) is characterized by the abrupt transition from the dry season with dominant northerly winds to the rainy season with prevailing southwesterly/southerly winds bringing substantial moisture^1,2^. Variations in the southwesterly/southerly flows and their moisture transport play a crucial role in the rainfall anomalies occurring over East Asia. A recent conceptual model study^3^ also showed that persistence and enhancement of low-level southerly flows of the EASM can be explained by moisture-advection feedback. Hence, better understanding of the low-level monsoon circulation and moisture source regions must improve our ability to predict the EASM rainfall. Although there have been several previous studies on moisture sources for summer monsoon rainfall over Asia, the dominant source regions for EASM precipitation are still debatable and assessed only based on model results. Conceptually, it has been emphasized that the EASM is accompanied with the substantial interhemispheric moisture transport from the SH to NH due to the strong cross-equatorial flow in the western part of the equatorial Indian Ocean and the equatorial Western Pacific^4–7^. However, modeling studies show diverse results. Lagrangian atmospheric water vapor tracer models show that the Tropical Indian Ocean^8,9^, Bay of Bengal and South China Sea^10–12^ and the North Pacific^13^ are identified as the dominant moisture source regions for EASM, whereas Eulerian approaches indicate the Northern Indian Ocean and North western Pacific^14,15^ contributing predominantly to precipitation during the EASM.

Atmospheric composition measurements can provide unique information about atmospheric transport and mixing processes. For monsoonal circulation studies, for instance, the rapid increase of atmospheric ^14^C-CO_2_ in the SH tropics during the boreal winter indicated influence of NH air masses carried south by a monsoon system^16^. The vertical transport of air masses into the lower stratosphere by the Indian Summer Monsoon (ISM) anticyclone has been investigated using satellite observations of water vapor^17^, ozone^18^, HCN^19^, and carbon monoxide^20^. However, the use of chemical tracers to unravel long-range meridional air mass transport and/or inter-hemispheric air mass exchange via strong low-level EASM flow has not previously been studied. Because a northward migration of the boreal summer ITCZ (Intertropical Convergence Zone) occurs on average to latitudes of 20°N over the Indian Ocean and adjacent land surface^21^, one generally cannot consider strong SH air mass intrusion being able to reach mid- latitudes (~33°N) in the EASM region after traveling more than 4,000 km. However, a chemical compound that is conservative on a time scale of the EASM seasonality, but has detectable regional gradients in its atmospheric mixing ratio, can be used as a tracer (see Supplementary Information (SI) text for more details)) to identify meridional transports of air masses.

To capture the imprint of meridional transport in the EASM, measurements of halogenated compounds with a large hemispheric gradient were considered. Specific criteria for a chemical tracer are the following: it should be (1) abundant in the atmosphere to assure a high signal-to-noise ratio; (2) have its dominant emission sources located in the NH; (3) have a chemical lifetime between one to ten years, and thus have clear latitudinal and/or NH-SH gradients which need to be 3 times higher than the measurement uncertainties. Among synthetic compounds, therefore mainly emitted/produced in the NH, hydrofluorocarbons (HFCs) have lifetimes similar to or longer than the time scale of inter-hemispheric exchange. In particular, HFC-152a with a lifetime of 1.5 years and HFC-134a with a lifetime of 14 years^22^ have been most widely used as refrigerants, aerosol propellants and foam-blowing agents^22^, replacing use of chloroflorocarbons (CFCs) and hydrochloroflorocarbons (HCFCs) in these applications, and thus their atmospheric abundances are relatively large compared to other new alternative HFCs^23^.

**Figure 1:**
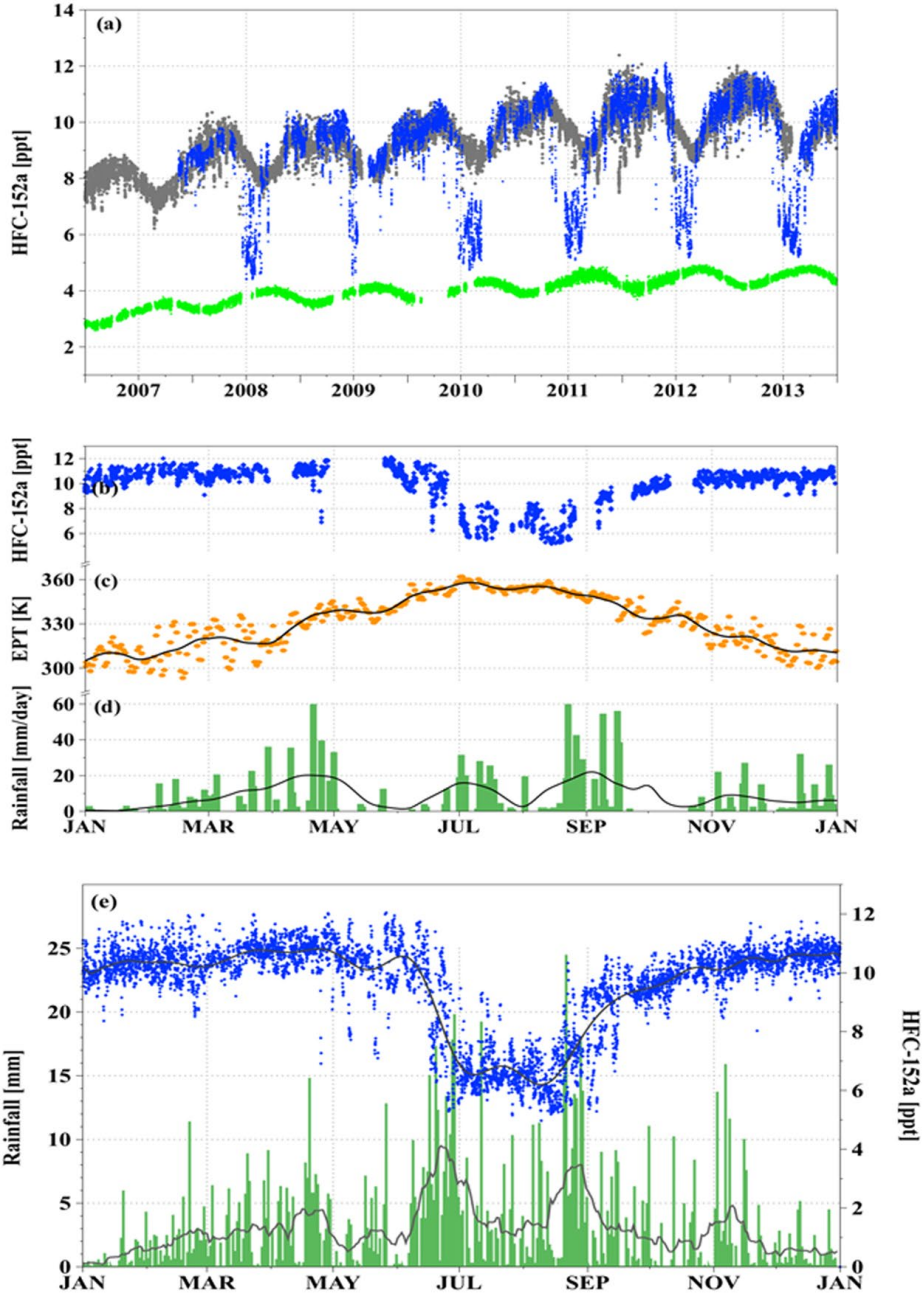
Periodic drawdowns of the HFC-152a concentrations in the EASM and corresponding equivalent potential temperature (EPT) and rainfall records. (**a**) HFC-152a background concentrations observed from 2008 to 2013 at the Gosan station [GSN, 33°N, 126°E] on Jeju Island, Korea are denoted by blue points. For comparison, the corresponding observations taken at the Mace Head station [MHD, 53°N, 10°W] in Ireland and at the Cape Grim station [CGO, 41°S, 145°E] in Australia are represented by gray and green points, respectively. (**b**) HFC-152a concentrations at Gosan for 2012, when continuous data were available without significant gaps for the entire EASM period, zoomed in from Fig. 1(a) as an example to show the annual pattern more in detail. (**c**) Equivalent potential temperature (EPT, K) at 850-hPa averaged over the southern part of South Korea [30–35°N, 125–130°E] for 2012. The black line represents a smooth fit using locally weighted least squares (“lowess”, smoothing window 0.085)). (**d**) Rainfall records measured by the Korean Meteorological Administration at Gosan for 2012 shown in green columns. The black line is a lowess fit with a smoothing window of 0.085. (**e**) The 6-year, hourly composite data of HFC-152a concentrations (blue) at Gosan that have been detrended with respect to the 2012 observations and one-day running means (gray line) are compared with climatological annual cycle of hourly rainfall from Gosan for the period 2008–2013 measured by KMA (bars) and 17-day running means (black line) (updated from Ha *et al*.^27^).

High-frequency atmospheric mole fractions of HFCs have been measured at the Gosan station^24^ (see SI text) on Jeju Island, South Korea since 2008 along with other major CFCs, HCFCs, PFCs, and SF_6_ to monitor their regional background levels and Asian continental outflows. Figure 1(a) shows the HFC-152a concentrations for 2008–2013 from the Gosan station in comparison to observations at Cape Grim in Australia (CGO, 41°S, 145°E), which represent the SH background mixing ratios, and to those at Mace Head in Ireland (MHD, 53°N, 10°W) considered as NH background mixing ratios. The time series show its global increases, a clear SH-NH gradient, and seasonal cycles due to summer time enhancements of the OH radical, the primary oxidant for HFCs (for HFC-134a, see SI Fig. S2). The most striking feature in the Gosan record is periodic drawdowns of the HFCs concentration occurring every summer, while during other months they are very consistent with the NH background level obtained at MHD. Note that the concentration drawdowns occur abruptly within a couple of days and the observed annual minima nearly reach concentrations measured in the mid-latitude SH (CGO). The drawdowns are much larger than the total measurement uncertainty of less than 1%, and three times larger than the OH-induced seasonality observed from an inland station at a similar latitude as Gosan (e.g., Shangdianzi site at 40°N)^25^. The periodic variations are also shown for very long-lived trace gases such as CF_4_, SF_6_ that do not react rapidly with OH (SI Fig. S3). Vertical dilution by free tropospheric air masses cannot be an explanation for the drawdowns given that no vertical gradients in HFCs mixing ratios were observed during the fourth and fifth HIAPER Pole-to-Pole Observations (HIPPO-4 and HIPPO-5) campaigns traversing the entire Pacific in June-early September 2011^26^. In contrast, the HIPPO results showed clear meridional gradients of HFC-152a from 30°S to 40°N below 1.5 km (SI Fig. S4) in good agreement with results from the Mauna Loa station (MLO, 19.5°N, 155.6°W) and CGO station^23^ and confirmed that the drawdowns at Gosan are similar to the tropical SH level. Therefore, the drawdown seasonality observed for HFC-152a concentrations at Gosan may be explained only by a large-scale low-level monsoonal circulation – a long-range, rapid transport of southern-hemispheric air crossing the tropics and reaching latitudes of 33°N, which has not been documented previously.

The summer drawdowns observed for HFCs are coincident with enhancements in equivalent potential temperature (EPT), which has been commonly used to identify EASM periods as an indicator of origin of air masses^1^. Minimum HFC concentrations approach SH background levels at EPT values above ~355 K, indicative of tropical air masses^1^ (Fig. 1(b,c)). However, EPT variations reflect only an integrated influence of EASM air masses, thus varying rather smoothly. The time series of daily rainfall amount in individual years (Fig. 1(d)) does not clearly identify EASM onset timing or direct tropical influence on air masses. Due to the rainfall fluctuations over a one-year period, multi-year composites of precipitation data are often used to diagnose the rainfall climatology, and the 6-year mean annual cycle of Korean rainfall between 2008 and 2013 (Fig. 1(e)) exhibits two major active rainfall periods called Changma and post-Changma, respectively^27^. Interestingly, for the HFC results, the six-year composite record is also sensitive to reveal two summertime drawdowns with a short break between them. The timing of the two dips also corresponds well to the two peaks in the climatological rainfall distribution. This concurrence implies that mechanistic driving forces of the EASM rainfall activity are associated with bringing in air depleted in HFCs, most likely from the tropical SH, and furthermore demonstrates the usefulness of HFCs as tracer for tracking air motions during the EASM, even though the details of how HFC variations and EASM activity are meteorologically related remain to be understood. The sensitivity of HFC mixing ratios to the large-scale air mass interactions is also demonstrated by a concentration drop occurring in late April, one to two months earlier than the major drawdowns corresponding to the EASM (see SI text and Fig. S5 for more details).

**Figure 2:**
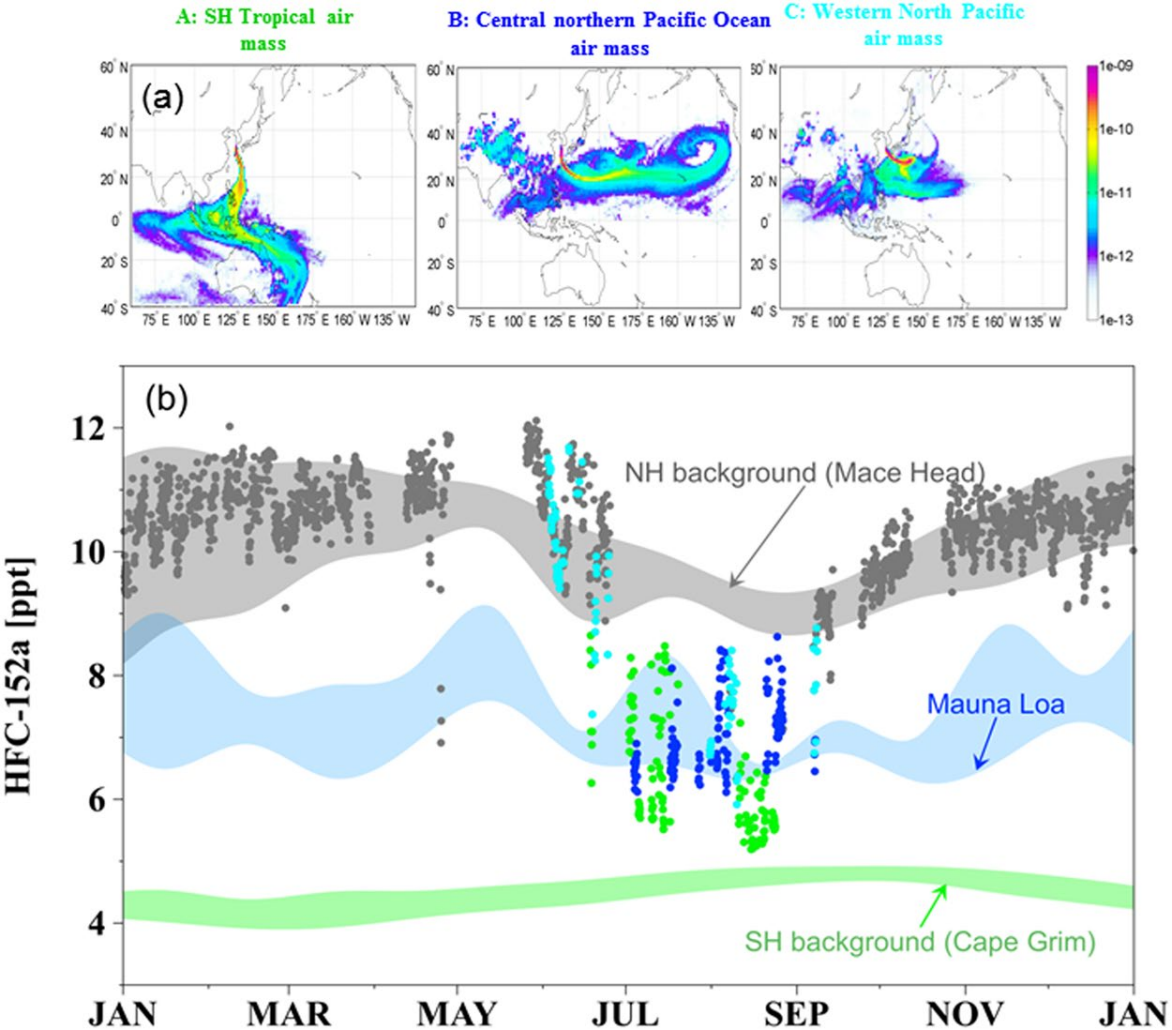
The HFCs drawdowns associated with a type of monsoonal air flow, indicating a previously undetected, abrupt and direct intrusion of SH air masses into the NH mid-latitudes. (**a**) Three types of maritime air masses presented by FLEXPART model: type A SH tropical air masses, type B central northern Pacific Ocean air masses, and type C air masses that stagnated in the western North Pacific area. Maps and trajectories are generated with MATLAB R2013a. (**b**) The HFC-152a data from Fig. 1(b) are replotted here in gray, green (type A), blue (type B), and light blue (type C). Gray and green shadings represent the 5^th^ to 95^th^ ranges of MHD and CGO observations for 2012, which are part of the 6-year records shown in Fig. 1(a). HFC-152a weekly flask measurements made at the MLO are represented by the light blue shading with the 5^th^ to 95^th^ percentiles.

A cluster analysis of air back-trajectories (SI text) shows that the EASM maritime air masses corresponding to the HFCs drawdowns over a 6-year period are grouped by their pathways and source origins into three typical types (Fig. 2(a)): tropical air masses moving very fast via the low-level southerly flow along the western boundary of the North Pacific subtropical high, passing through the south China sea (type A); air masses crossing the central northern Pacific Ocean due to a westward expansion of the large-scale North Pacific anticyclone (type B); air masses that originated from the western North Pacific area and stagnated from the origin up to the East Asian region (type C). Type A accounts for 39.2% of the maritime air, B for 32.2% and C for 28.6%, showing significant contribution of type A to the maritime air masses. Each HFC measurement was associated with a type of monsoonal air flow (Fig. 2(b)). HFC concentrations typically lower than measured at MLO are all corresponding to tropical air masses (type A) (SI text and Fig. S6); in fact, about 70.5% of the type A data are closer to values measured well into the SH at CGO. The HFC-152a concentrations in air masses originating from the central northern Pacific Ocean (type B) are consistent with results from MLO^23^. HFC concentrations in type B air masses reflect the connections between the EASM and the North Pacific anticyclone near Hawaii^28^, of which intensity variation and/or migration may be signified by a half month break of EASM observed in July both from the HFC time series and the 6-year rainfall climatology (Fig. 1(e)). HFC concentrations in type C air masses are distributed over a wide range from NH background to MLO values, which potentially indicates competitive interactions between tropical air masses versus cold Okhotsk marine air masses that often develop over East Asia in early summer^1^ and/or influence of polluted Asian continental air masses. Thus, the HFC drawdown onset and duration are most likely determined by a strong and abrupt intrusion of tropical air masses with substantial SH.

**Figure 3:**
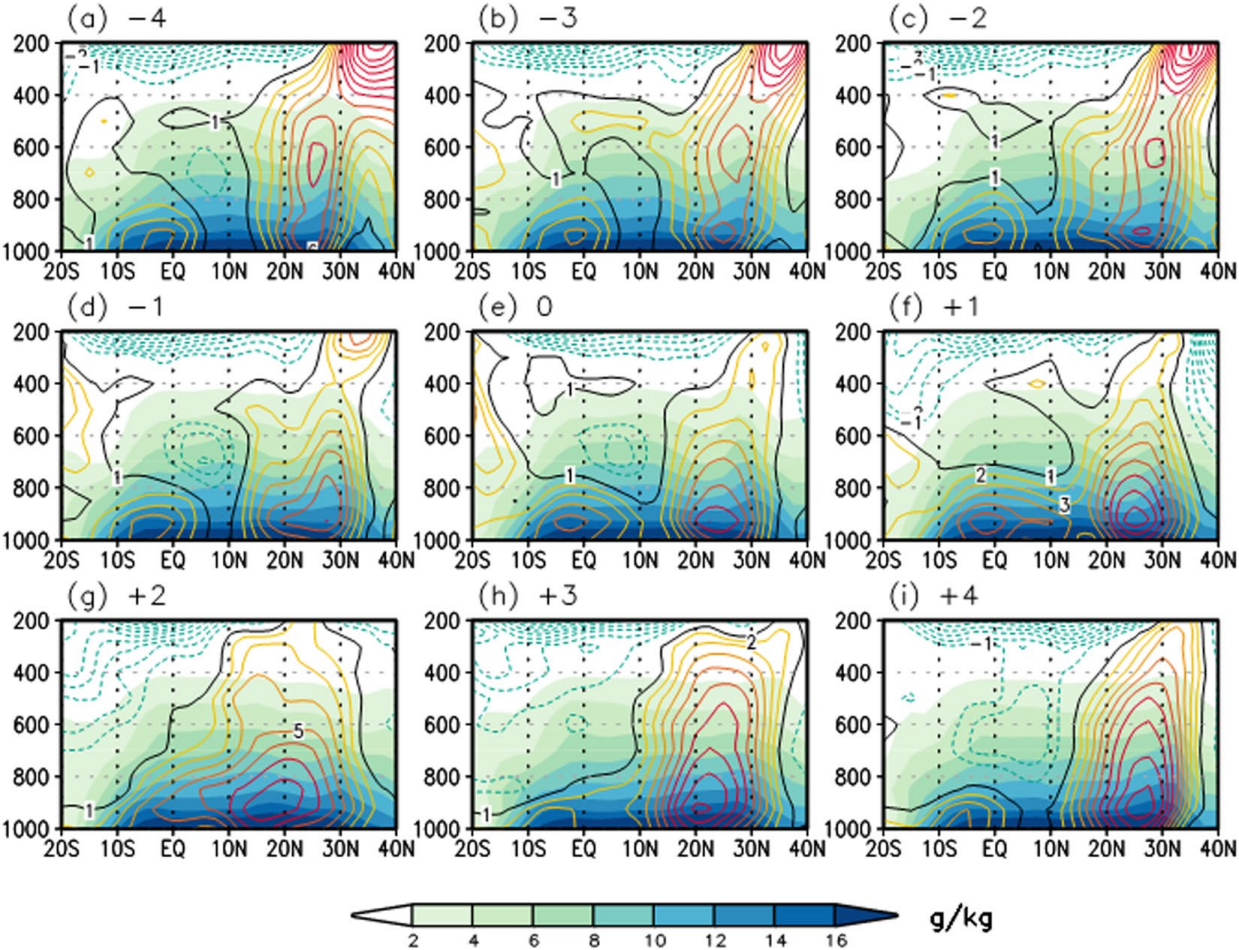
Northward migration of Low-level southerly accompanying moisture transport from the SH into East Asia. The latitude-pressure cross sections of daily meridional wind (contour, m s^−1^) and specific humidity (shading, g/kg) along the EASM sector (125°–130°E) from 4 days before (**a**) and after (**i**) onset of HFC-152a drawdown averaged over 2008–2013. The contour interval of meridional wind is 1 m s^−1^. The data were obtained from the National Centers for Environmental Prediction-U.S. Department of Energy reanalysis II. Figures are generated with GrADS 2.0.1.oga.1.

We further investigated the relative contributions of the three air mass types to the EASM rainfall using the Korean Meteorological Administration (KMA) records of precipitation at Gosan, and found that the largest rainfall in the EASM occur most often when tropical air masses (type A) dominate over the region, accounting for more than 51% of the JJA precipitation during 2008 to 2013 (SI Fig. S7(a)). Specific humidity (g/kg), a measure of precipitable water^8^, in each trajectory during the six hours before arrival at Gosan gave consistent results; the specific humidity observed from type A trajectories showed the most significant potential for moisture rainout (SI Fig. S7(b,c)). This considerable moisture transport of tropical air masses with SH character during the EASM is also consistent with the concurrence of the two dips of HFCs and the two active rainfall peaks in the climatological rainfall distribution (Fig. 1(e)). The six-year composite latitude-pressure cross sections of daily meridional wind^29^ and specific humidity^29^ along the EASM sector further demonstrate the intensification of low-level southerly flow and accompanied moisture transport from the SH during and after the onset of HFC-152a drawdown (Fig. 3(f–i)) compared to those before the onset (Fig. 3(a–d)). Before the onset, there is the weak low-level northerly wind between 5° and 15°N due to the separate smaller meridional cell in the SH from the major local Hadley cell in the NH. However, the center of low-level southerly located at 5°S around 4 days before the onset (Fig. 3(a)) migrates northward (Fig. 3(b–d)). Note that the northward migration of the center of southerly in the SH generally starts four or three days before the onset. During the onset, the two cells are combined and the continuous low-level southerly can transport the SH moist and clear air into East Asia (Fig. 3(e)). After the onset, the local Hadley circulation is intensified significantly, particularly two days after the onset (Fig. 3(g)), attributable to the positive feedback between the low-level southerly wind associated with divergence from the SH tropics and subsequent latent heat release of transported moisture. It is consistent with recent studies^2,30^ presenting the importance of the feedback of condensational heating induced by monsoon rain, which acts to largely enhance low-level flows of EASM as well as further in forming and maintaining the EASM. Our understanding in the detail physical mechanism on the abrupt intrusion of the SH air mass into the EASM region triggering the EASM onset remains still limited. In addition, it is a challenge for the state-of-the-art numerical models to capture the abrupt onset of summer monsoon on a short timescale of a couple of days^31–33^. Nonetheless, this study suggests that the combination of direct chemical tracer record and a back-trajectory model with physical meteorological variables offer a better way to identify moisture source regions for monsoon rainfall and to advance in understanding of large-scale zonal and meridional air movement associated with the EASM.

## Methods

High-precision and high-frequency measurements of halogenated compounds introduced in this study, were made continuously every two hours from 2008 to 2013 using a gas chromatograph-mass spectrometer coupled with an online cryogenic pre-concentration system (“Medusa”)^34^ within the AGAGE program. Analytical uncertainties of the measurements are 0.2% (RSD). Figures 1(a) and S2 show time series of HFC-152a and HFC-134a for 2008–2013, respectively, from the Gosan station in comparison to observations at CGO and to those at MHD. Figure 1(a) shows the HFC-152a concentrations after removing the regional pollution events from the original observations obtained at Gosan. The calculation algorithm is given in O’Doherty *et al*.^35^. The corresponding time series for HFC-134a is also shown in Fig. S2. Weekly flask measurements of HFC-152a and HFC-134a at MLO in Figs 2(b) and S2, respectively, have been taken since 2008. The data are provided from the Global Monitoring Division of the Nationals Oceanic and Atmospheric Administration’s Earth System Research Laboratory (NOAA/ESRL/GMD) (ftp://ftp.cmdl.noaa.gov/hats/hfcs/).

## Electronic supplementary material

Supplementary Information
