# Supplementary material for: Chemical evidence of inter-hemispheric air mass intrusion into the Northern Hemisphere mid-latitudes
Source: Sci Rep. 2018 Mar 16;8:4669. doi: 10.1038/s41598-018-22266-0 (PMC5856755; doi:10.1038/s41598-018-22266-0)
Supplement: Supplementary file 1 — Supplementary Information [file 41598_2018_22266_MOESM1_ESM.pdf]

Supplementary Information

Chemical evidence of inter-hemispheric air mass intrusion into the Northern Hemisphere mid-latitudes

S. Li[1], S. Park[1,2*], J.-Y. Lee[3], K.-J. Ha[3,4], M.-K. Park[1], C. O. Jo[1], H. Oh[4], J. Mühle[5], K.-R. Kim[6], S. A. Montzka[7], S. O'Doherty[8], P. B. Krummel[9], E. Atlas[10], B. R. Miller[7], F. Moore[7], R. F.Weiss[5], S. C. Wofsy[11]

[1]Kyungpook Institute of Oceanography, College of Natural Sciences, Kyungpook National University, Daegu, South Korea

[2]Department of Oceanography, School of Earth System Sciences, Kyungpook National University, Daegu, South Korea

[3]Research Center for Climate Sciences, Pusan National University, Busan, South Korea

[4]Division of Earth Environmental System, Department of Atmospheric Sciences, Pusan National University, Busan, South Korea

[5]Scripps Institution of Oceanography, University of California, San Diego, La Jolla, CA, USA

[6]GIST College, Gwangju Institute of Science and Technology, Gwangju, South Korea

[7]Earth System Research Laboratory, NOAA, Boulder, CO, USA

[8]School of Chemistry, University of Bristol, Bristol, UK

[9]Oceans and Atmosphere Flagship, CSIRO, Aspendale, Victoria, Australia

[10]Rosenstiel School of Marine and Atmospheric Science, University of Miami, Miami, USA

[11]School of Engineering and Applied Sciences, Harvard University, Cambridge, MA, USA

*Correspondence to: sparky@knu.ac.kr.

The supplementary information contains supplementary text, figures S1-S7.

Gosan station: The Gosan station (GSN, 33.25°N, 126.19°E, Jeju Island, Korea) is located on the boundary between the Pacific Ocean and the Asian continent, and experiences distinct seasonal wind and weather patterns characterized by warm wet East Asian Summer Monsoon (EASM) and cold dry winter (Fig. S1), and thus provides an ideal place to monitor both Asian continental outflows and maritime air mass intrusion.

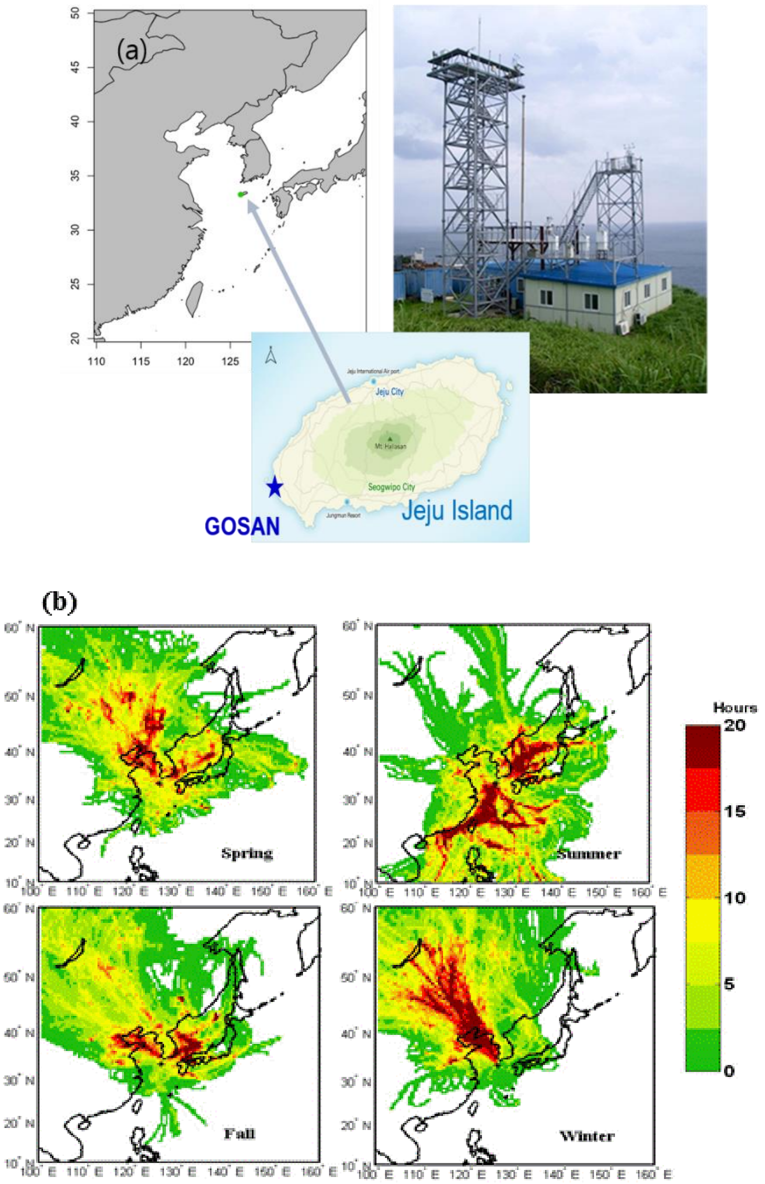

Figure S1. **(a)** The Gosan AGAGE (Advanced Global Atmospheric Gases Experiment) station is located atop a 72-m cliff on the remote south-western tip of Jeju Island, 100 km south of the Korean peninsula, allowing for monitoring of long-range transport from the surrounding region. **(b)** The residence time analysis using 5-day back-trajectories arriving at the Gosan station for year 2008 made by HYSPLIT 4.8 model shows that major air masses dominating the region vary seasonally: predominant northwesterly and northeasterly continental outflows from fall through spring versus air flows of clean air directly from the Pacific in summer and from northern Siberia in winter. Maps and plots are generated with MATLAB R2013a.

Selection of chemical tracers: Criteria for chemical tracers to capture the imprint of meridional transport are: the chemical species should be (1) abundant in the atmosphere to assure a high signal-to-noise ratio; (2) have its dominant emission sources located in the NH; (3) have a chemical lifetime between one to ten years, and thus show clear latitudinal and/or NH-SH gradients in its atmospheric mixing ratio. Among synthetic compounds, therefore mainly emitted/produced in the NH, hydrofluorocarbons (HFCs) have lifetimes similar to or longer than the time scale of inter-hemispheric exchange. In particular, HFC-134a with a lifetime of 14 years[1] and HFC-152a with a lifetime of 1.6 years[1] have been most widely used among HFCs since their introduction in the 1990s. Their major uses are as refrigerants, aerosol propellants and foam-blowing agents[1], replacing use of chlorofluorocarbons (CFCs) and hydrochlorofluorocarbons (HCFCs) in these applications.

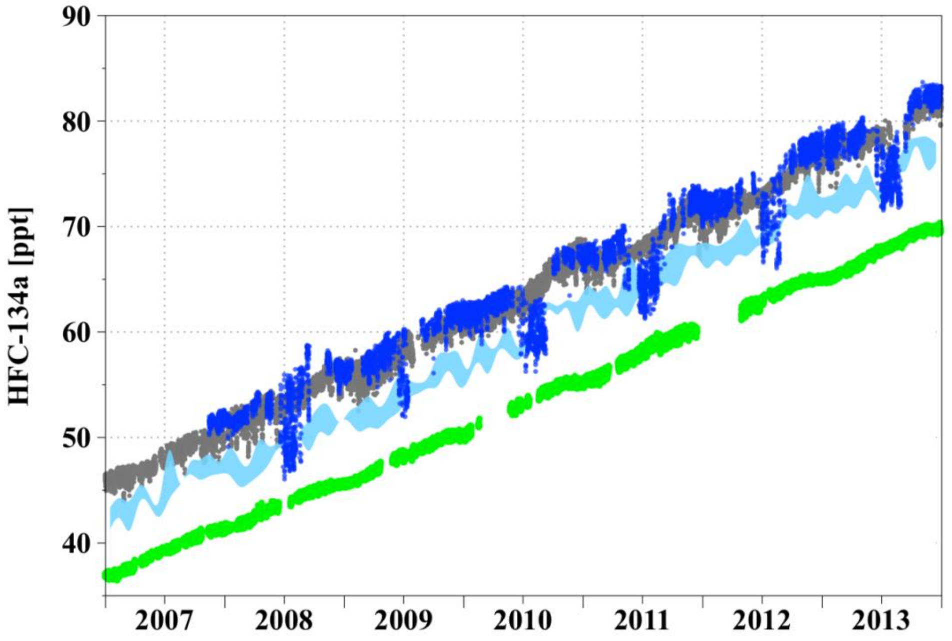

Figure S2. Background concentrations of HFC-134a observed from 2008 to 2013 at the Gosan station. For comparison, the corresponding observations at the Mace Head station and at the Cape Grim station are represented by gray and green points, respectively. The flask sample data obtained from the Mauna Loa station are denoted by light blue shading with the 5th to 95th percentiles.

***Influences of OH seasonality and vertical dilution on the HFCs drawdowns**:* We found the same drawdown features in the time series of CF_4 and SF_6 that have much longer atmospheric lifetime than HFCs, and thus are not affected by seasonal variations of the OH radical concentrations. Thus, their drawdowns cannot be the results of OH seasonality (Fig. S3).

Vertical dilution by free tropospheric air masses is also an unlikely explanation for the drawdowns given the fact that we cannot detect a clear vertical gradient up to the altitude of 8 km in HFC-152a mixing ratio observed from the fourth and fifth HIAPPER Pole-to-Pole Observations (HIPPO-4 and HIPPO-5) (http://hippo.ornl.gov/dataaccess) during the boreal summer (Fig. S4).

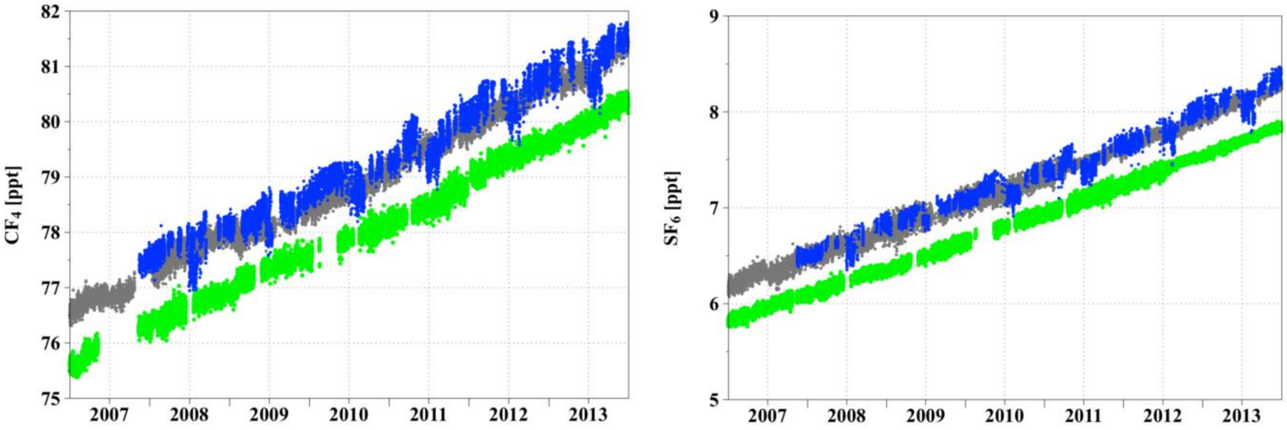

Figure S3. Time series of CF_4 and SF_6 observed from 2008 to 2013 at the Gosan station. Same symbols as in Fig. S2.

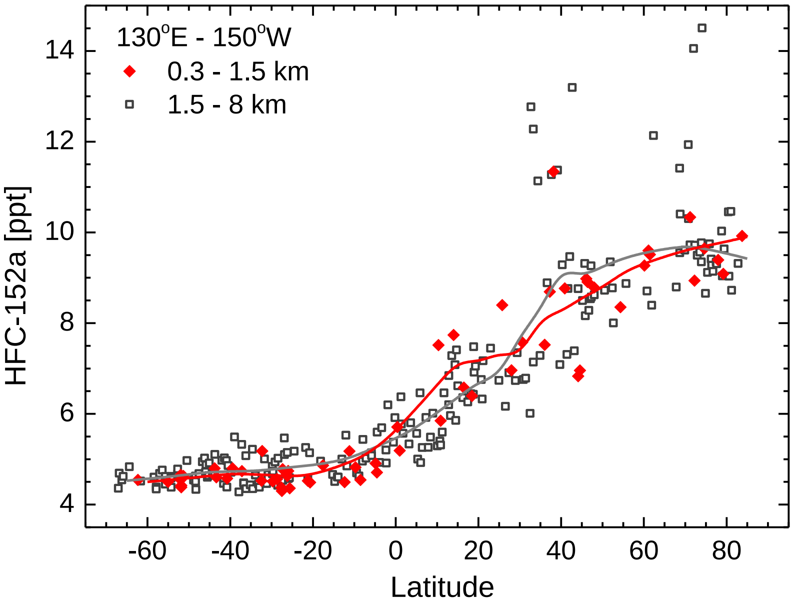

Figure S4. Meridional gradients of HFC-152a sampled between 0.3 and 1.5 km (filled red diamonds) and between 1.5 and 8 km (empty gray squares) during the HIPPO-4 (June 14th to July 10th, 2011) and HIPPO-5 (Aug. 18th to Sept. 6th, 2011) traversing over the western boundary and the center of the Pacific. The solid lines denote Lowess fits with the smoother span (*f*) =*0.25* for each data set.

Dropdowns of HFCs in late April: The climatological summary[2] of the regional onset dates of Asian monsoon shows that the earliest onset of the monsoon arrives first at the central Indochina Peninsula Fin late April and early May, and then the monsoon system advances northward along the BOB (Bay of Bengal) and the south China sea, finally arriving in the East Asian region in June. The dropdowns of the HFC mixing ratio observed at Gosan in late April coincided with the ISM onset dates defined by zonal wind shear index[3] (Fig.S5). Given the fact that the ISM is initiated by development of an active BOB cyclone[2], the HFC dropdowns in spring seem to be a fingerprint of coherent propagation of the accelerated low-level westerly and significant convection activity over the BOB, thereby indicating a tele-connected perturbation of the East Asian air masses by the mechanism(s) controlling the ISM and/or the subsequent convective motion.

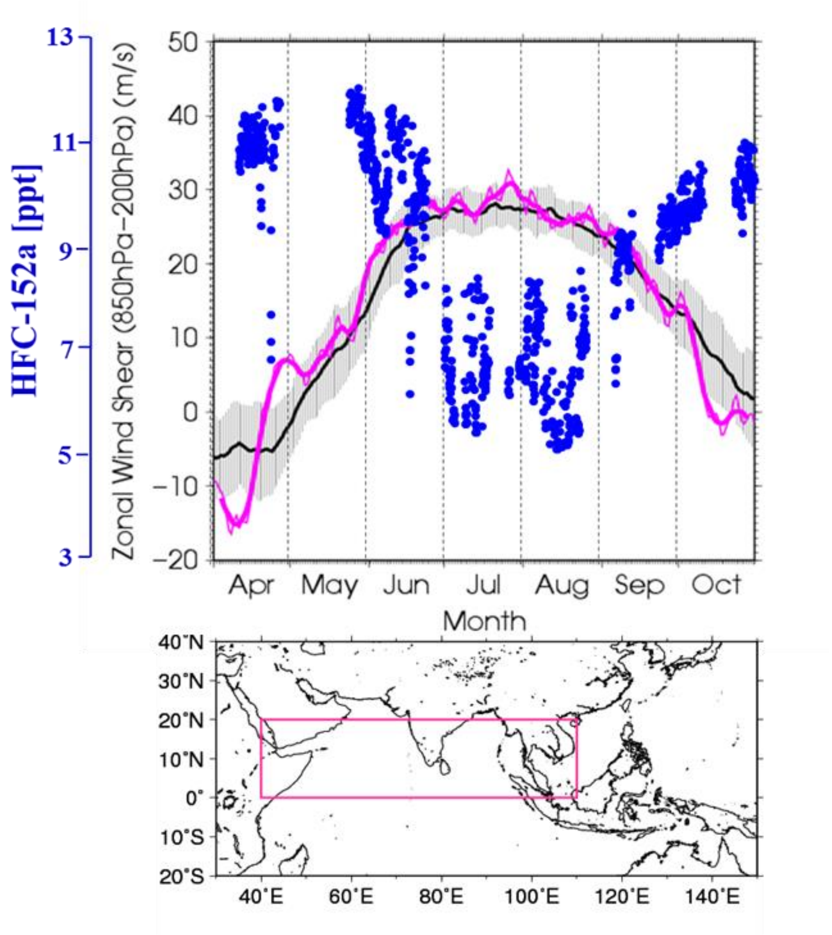

Figure S5. Based on the time-series representation of the zonal wind shear index averaged over the North Indian Ocean and southern Asia (shown by the rectangle on the bottom: equator - 20°N, 40°E - 110°E) between 200hpa and 850hpa for 2012 (Japan Meteorological Agency)(http://ds.data.jma.go.jp/tcc/tcc/products/clisys/ASIA_TCC/monsoon_index.html), the HFC-152a measurements at Gosan for 2012 were superimposed on the zonal wind shear index figure and denoted by blue circles. The zonal wind shear index is calculated after Webster

and Yang (1992). The thick and thin pink lines indicate seven-day running mean and daily mean values, respectively for 2012. The black line denotes the normal (i.e., the 1981 - 2010 average), and the gray shading shows the range of the standard deviation calculated for the time period of the normal.

Trajectory calculations: To further examine the relationship between HFC variations observed during the EASM periods and air-mass transport pathways and origins, we analyzed 5-day back trajectories of air masses arriving at Gosan in June, July, and August (JJA) from 2008 to 2013. The back trajectories were calculated using the HYSPLIT 4.8 modeling[4] with meteorological output fields from the National Center for Environmental Prediction (NCEP) Global Data Assimilation System (GDAS; http://www.ready.noaa.gov/archives.php). These data are provided on a 1° latitude-longitude grid and every 3 hour. Statistics of air clusters were calculated for the 4078 back trajectories.

When we examined the HYSPLIT model runs in comparison with a particle dispersion back trajectory model (i.e., FLEXPART), we found the results at 500-m altitude consistent between the two models. So we used the single particle trajectory model since it has been proven in both computational readiness and actual use for the cluster analysis[5]. Then, the resulting three types of air clusters were presented by using the FLEXPART software in Fig. 2(a), because it illustrates the air distribution of each cluster in a more realistic way.

Sensitivity of HFCs tracer: The HFCs measurements were associated with a type of monsoonal air flow (Figs. 2(a), (b) and Fig. S6). Tropical air masses (type A in Fig. 2(a)) are typically moving very fast via the low-level southerly flow along the western boundary of the western North Pacific (WNP) subtropical high, passing through the South China Sea. As expected, most of the HFCs measurements corresponding to tropical air masses (type A: Fig.S6(c)) are lower than those measured at Mauna Loa (MLO) and closer to SH background values while some measurements, especially in July, show higher values than those for most of type A air masses. We found that those measurements correspond to the air masses that originated from the tropical SH, but curved over the east coast of China (Fig. S6(b)), where intensive industrial activity for HFCs production and consumption occurs, and therefore, reflect the influence of regional pollution plume on the low-level tropical air masses. This demonstrates the HFCs tracer signals are sensitive not only to air mass origins, but also to pathways.

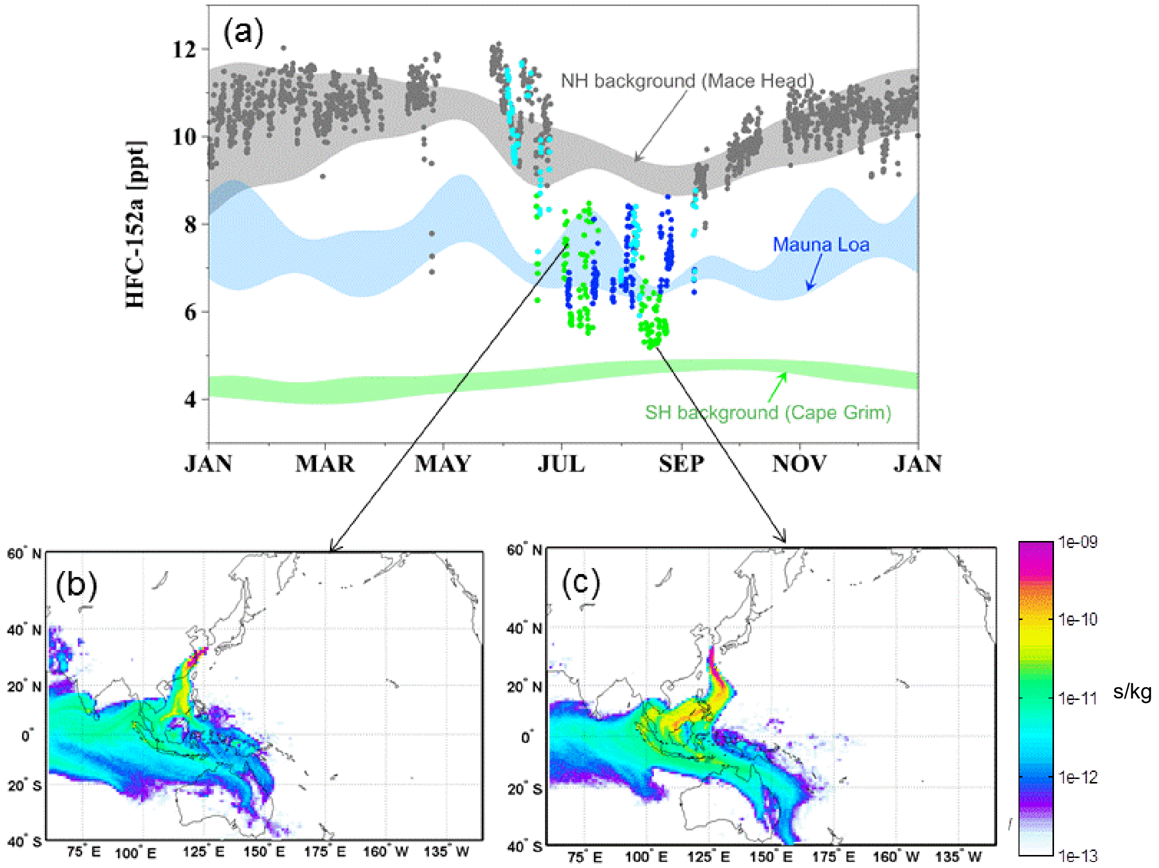

Figure S6. **(a)** Same descriptions as in Fig. 2(b). **(b)** The HFC-152a data associated with tropical air masses group (type A) passing over the east coast of China are closer to MLO's values, but **(c)** most of data corresponding to tropical air masses passing through the South China Sea are lower than MLO's level and closer to SH background values. Maps and trajectories are generated with MATLAB R2013a.

Relative contributions of the three air mass types to the EASM rainfall: Precipitation amount for each air mass type was estimated by integrating the precipitation amounts observed for all air masses with trajectories categorized into the corresponding type arrived at Gosan during 2008 to 2013. The relative contribution of each air mass type was then derived by considering total EASM rainfall during JJA.

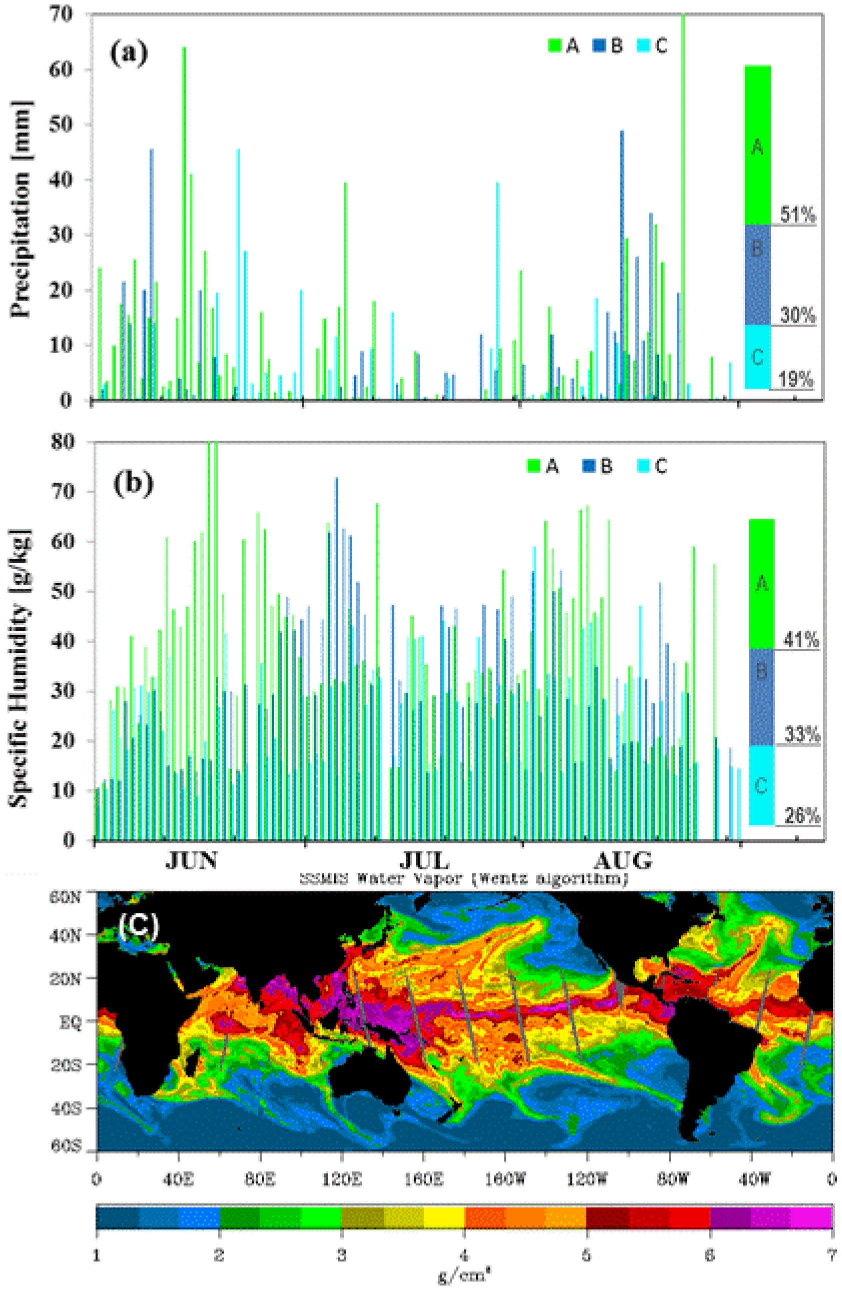

Figure S7. **(a)** Total precipitation amounts (mm) were estimated by integrating the precipitation amounts observed for each different monsoonal maritime air mass of types A, B, and C arriving at Gosan during 2008 to 2013 based on the Korean Meteorological Administration records and the relative contributions in percentage to 6-year of the JJA EASM

rainfall. The type A air masses are responsible for ~50% of the EASM precipitation. **(b)** Integrated specific humidity (g/kg) obtained from NOAA NECP/NCAR Reanalysis data (4-times daily, 2.5° x 2.5° global grid) for all the trajectories of each air mass type during the six hours before arrival at Gosan. Note the large specific humidity associated with moisture rainout was observed from the type A trajectories. **(c)** Water vapor transport image on the 10th of June in 2011 taken from NOAA Special Sensor Microwave Imager Sounder (2-times daily, 0.5 x 0.5°) (https://www.esrl.noaa.gov/psd/psd2/coastal/satres/data/images/archive/2xdaily/global_iwv/11161_wvp_am_gl.png).

References

1. Carpenter, L. J. & Reimann,S. Ozone-depleting substances(ODSs) and related chemicals, in Scientific Assessment of Ozone Depletion:2014, Global Ozone Research and Monitoring Project in Rep. 55, Chap.**1**, 1-108, *WMO*, Geneva, Switzerland (2014).
2. Ding, Y. & Chan, J. C. L. The East Asian summer monsoon: an overview. *Meteorol. Atmos. Phys*. **89**, 117-142 (2005).
3. Webster, P. J. & Yang, S. Monsoon and ENSO: Selectively Interactive Systems. *Quart. J. Roy. Meteor. Soc*., **118**, 877-926 (1992).
4. R. Draxler, G. D. Hess, NOAA technical memorandum ERL ARL-224: Description of the HYSPLIT_4 modeling system (2004). Available at www.arl.noaa.gov/documents/reports/arl-224.pdf
5. An, Z. S. *et al*., Glacial-interglacial Indian summer monsoon dynamics. *Science*, **333**, 719-723 (2011).
